# EVALUATING A PHONE-BASED SUPPORT PROGRAM TO ADDRESS LONELINESS: LESSONS LEARNED FROM PARTICIPANTS AND STAFF

**DOI:** 10.1093/geroni/igac059.616

**Published:** 2022-12-20

**Authors:** Carla Perissinotto, Ashwin Kotwal, Soe Han Tha, Katrina Hough, Bri Matusovsky

**Affiliations:** University of California, San Francisco, San Francisco, California, United States; University of California, San Francisco, San Francisco, California, United States; University of California, San Francisco, San Francisco, California, United States; University of California, San Francisco, San Francisco, California, United States; University of California, San Francisco, San Francisco, California, United States

## Abstract

**Introduction:**

Phone-based support programs are increasingly being used as a solution for those experiencing loneliness. However, less is known about what aspects of these support programs are most helpful to participants and even less known about the staff and volunteer who work in these programs.

**Methods:**

Mixed methods structured surveys of participants (N=247 baseline, N=147 follow-up), and in-depth qualitative interviews of both participants (N=15, and staff=23).

**Results:**

We present the results from the qualitative interviews with a focus on what barriers and facilitators are to implementing a phone-based support intervention, particularly for older adults experiencing loneliness. Preliminarily, 77% of staff and volunteers felt more connected themselves through their role in the phone-support program. 100% of staff also believe the callers feel less lonely as a result of using the line, and 80% feel they create a meaningful relationship with callers. Themes included and overall sense of need for expansion of these services, while better understanding the optimal length and frequency of calls. Consistent with staff responses, amongst callers, 90% fell more socially connected because they use the telephone support line. Similarly, to staff and volunteers, participants felt their needs were met during calls, but wished the length of calls could be flexible. This demonstrates that the line is beneficial to both participants and staff. There is high satisfaction on the quality of the calls and the connections made, but emerging themes suggest a need to scale services to meet demand at all hours and allow for flexibility in length of calls.

